# Peritoneal cytology in the surgical evaluation of gastric carcinoma

**DOI:** 10.1038/sj.bjc.6690081

**Published:** 1999-02

**Authors:** N Hayes, J Wayman, V Wadehra, D J Scott, S A Raimes, S M Griffin

**Affiliations:** The Northern Oesophago-Gastric Cancer Unit, The Royal Victoria Infirmary, Queen Victoria Road, Newcastle upon Tyne, NE1 4LP, UK; Institute of Pathology, The Royal Victoria Infirmary, Queen Victoria Road, Newcastle upon Tyne, NE1 4LP, UK

**Keywords:** gastric cancer, peritoneal cytology, prognosis

## Abstract

Many patients undergoing surgery for gastric carcinoma will develop peritoneal metastases. A method to identify those patients at risk of peritoneal recurrence would help in the selection of patients for adjuvant therapy. Peritoneal cytology has received little attention in the West, but may prove a useful additional means of evaluating patients with gastric cancer. The aims of this study were to evaluate sampling techniques for peritoneal cytology in patients with gastric cancer, to assess the prognostic significance of free peritoneal malignant cells and to discover the effect of the operative procedure on dissemination of malignant cells. The study is based on 85 consecutive patients undergoing surgical treatment of gastric cancer and followed up for 2 years or until death. Peritoneal cytology samples were collected at laparoscopy, and at operation prior to resection by intraperitoneal lavage and serosal brushings. After resection, samples were taken by peritoneal lavage, imprint cytology of the resected specimen and post-operatively by peritoneal irrigation via a percutaneous catheter. Malignant cells were diagnosed by two independent microscopists. Preoperative peritoneal lavage yielded malignant cells in 16 out of 85 cases (19%). The yield of free malignant cells was increased by using serosal brushings (by four cases) and imprint cytology (by two cases); all of the cases had evidence of serosal penetration. One serosa-negative case exhibited positive cytology in the post-resection peritoneal specimen in which the preresection cytology specimen was negative. Survival was worse in the cytology-positive group (χ^2^ = 25.1; *P* < 0.0001). Among serosa-positive patients, survival was significantly reduced if cytology was positive, if cases yielded by brushings and imprint cytology were included (log-rank test = 8.44; 1 df, *P* = 0.004). In conclusion, free peritoneal malignant cells can be identified in patients with gastric cancer who have a poor prognosis; the yield can be increased with brushings and imprint cytology in addition to conventional peritoneal lavage. Evaluation of peritoneal cytology by these methods may have a role in the selection of patients with the poorest prognosis who may benefit most from adjuvant therapy. © 1999 Cancer Research Campaign


*British Journal of Cancer* (1999) **79**(3/4), 520–524
© 1999 Cancer Research Campaign
Article no. bjoc.1998.0081

### Peritoneal cytology in the surgical evaluation of gastric carcinoma

**N Hayes[1], J Wayman[1], V Wadehra[2], DJ Scott[2], SA Raimes[1] and SM Griffin[1]**

[1]The Northern Oesophago-Gastric Cancer Unit and [2]Institute of Pathology, The Royal Victoria Infirmary, Queen Victoria Road, Newcastle upon Tyne NE1 4LP, UK

**Summary** Many patients undergoing surgery for gastric carcinoma will develop peritoneal metastases. A method to identify those patients at risk of peritoneal recurrence would help in the selection of patients for adjuvant therapy. Peritoneal cytology has received little attention in the West, but may prove a useful additional means of evaluating patients with gastric cancer. The aims of this study were to evaluate sampling techniques for peritoneal cytology in patients with gastric cancer, to assess the prognostic significance of free peritoneal malignant cells and to discover the effect of the operative procedure on dissemination of malignant cells. The study is based on 85 consecutive patients undergoing surgical treatment of gastric cancer and followed up for 2 years or until death. Peritoneal cytology samples were collected at laparoscopy, and at operation prior to resection by intraperitoneal lavage and serosal brushings. After resection, samples were taken by peritoneal lavage, imprint cytology of the resected specimen and post-operatively by peritoneal irrigation via a percutaneous catheter. Malignant cells were diagnosed by two independent microscopists. Preoperative peritoneal lavage yielded malignant cells in 16 out of 85 cases (19%). The yield of free malignant cells was increased by using serosal brushings (by four cases) and imprint cytology (by two cases); all of the cases had evidence of serosal penetration. One serosa-negative case exhibited positive cytology in the post-resection peritoneal specimen in which the preresection cytology specimen was negative. Survival was worse in the cytology-positive group ($\chi^2 = 25.1$; $P < 0.0001$). Among serosa-positive patients, survival was significantly reduced if cytology was positive, if cases yielded by brushings and imprint cytology were included (log-rank test = 8.44; 1 df, $P = 0.004$). In conclusion, free peritoneal malignant cells can be identified in patients with gastric cancer who have a poor prognosis; the yield can be increased with brushings and imprint cytology in addition to conventional peritoneal lavage. Evaluation of peritoneal cytology by these methods may have a role in the selection of patients with the poorest prognosis who may benefit most from adjuvant therapy.

**Keywords:** gastric cancer; peritoneal cytology; prognosis

More patients with adenocarcinoma of the stomach are presenting at an earlier stage (Sue-Ling et al, 1993), yet most who undergo surgery with curative intent will ultimately die of recurrent disease (Papachristou and Fortner, 1981). Relook laparotomy and post-mortem studies have demonstrated that the majority of patients who have recurrent disease will have peritoneal metastases (Gunderson and Sosin, 1982). Peritoneal recurrence presumably stems from transcoelomic dissemination of malignant cells in the pre- or perioperative period (Sugarbaker et al, 1988). Despite this, the role of peritoneal cytological evaluation in predicting failure of gastric cancer resections has attracted little interest in the West. Japanese workers, by contrast, place great store in the determination of peritoneal cytology status in patients undergoing surgery for gastric cancer (Kaibara et al, 1987; Boku et al, 1990), and have even used peritoneal cytology to examine the effect of adjuvant chemotherapy (Iitsuka et al, 1979).

Open lavage, performed immediately before tumour resection, is a crude but simple technique for sampling potentially free cells within the peritoneal cavity. A previous pilot study at this institution demonstrated that less than one-third of patients with advanced gastric cancer had positive peritoneal cytology at the time of operation (Murphy et al, 1993). This is significantly fewer cases than the proportion which might be expected to die with peritoneal metastatic recurrent disease; historically, up to 80% (Gunderson and Sosin, 1982). This underdetection may be due to the insensitivity of the sampling technique, or it may be that the process of surgical resection itself encourages the dissemination of malignant cells (Cunliffe and Sugarbaker, 1989). If detection of free malignant cells in the peritoneal cavity is to be used to identify patients at risk of peritoneal recurrence and prove useful in the selection of patients for adjuvant therapy, its technique needs critical evaluation.

The aims of this study were to evaluate the technique of peritoneal lavage with alternative peritoneal cytological sampling strategies, to discover whether surgical resection can be shown to initiate peritoneal dissemination of tumour cells and, hence, explain the unexpectedly low yield of preresection peritoneal lavage, and lastly to determine the prognostic significance of peritoneal cytological evaluation.

Ethical approval for this study was granted by the Joint Ethics Committee of Newcastle District Health Authority and the University of Newcastle upon Tyne.

*Received 2 February 1998*
*Revised 1 May 1998*
*Accepted 9 June 1998*

*Correspondence to:* SM Griffin, The Royal Victoria Infirmary, Queen Victoria Road, Newcastle upon Tyne NE1 4LP, UK

### PATIENTS AND METHODS

### Patients

Patients undergoing elective surgery for adenocarcinoma of the stomach were eligible for this study. Recruitment began in April

1992 and took place in four surgical units within the former Northern Region (see Acknowledgements). All participating patients were interviewed by the principal author before providing written, informed consent.

### Intraoperative staging

At operation, gastric cancers were staged for local, nodal and metastatic spread. Any apparent breach of the gastric serosal layer was mapped using a clear plastic grid (Opsite Flexigrid, Smith and Nephew, Hull, UK), and the surface area was calculated. The extent of surgical resection was not controlled by the study, but left to the discretion of the surgeon involved in each case.

### Specimen collection

### *Preresection*

***Peritoneal washing*** Immediately on opening the abdomen, 100 ml of warm saline was instilled onto the serosa overlying the tumour. The fluid was then aspirated from the abdominal recesses, including the pouch of Douglas. If ascites was present, this fluid was aspirated instead of performing a wash. A minimum of 80% (80 ml) retrieval was required for these and all other washings to be regarded as adequate for analysis. In practice, this was achievable in all cases.

***Laparoscopic washing*** Patients undergoing laparoscopic staging before open surgery were included in this study. Saline lavage was performed at the time of laparoscopy using a combined suction/irrigation rigid catheter passed through a 5 mm epigastric port. Saline (100 ml) was instilled over the anterior surface of the stomach and aspirated under direct vision.

***Peritoneal brushings*** These were obtained by lightly sweeping the serosa overlying the tumour with a sterilized brush (CerviBrush+, CellPath, Hemel Hempstead, UK). Brushings were made over the entire area of suspected serosal penetration using a single brush sweeping the area four times. The slide was then smeared onto a series of slides for immediate fixation.

### *Post-resection*

***Imprint cytology*** When palliative or curative resection was possible, the specimen was handed to an assistant and slides coated with poly-L-lysine (Sigma, Poole, UK) were pressed against the serosa overlying the tumour and immediately fixed.

***Peritoneal washing*** A second washing was obtained after completion of the surgical manoeuvres, but before any therapeutic lavage (e.g. with tetracycline solution) was used. The saline lavage was particularly directed at the gastric bed and pedicles where resection had taken place. This procedure was not included if the patient had only an exploratory laparotomy.

***Peritoneal irrigation*** At the end of the operation, a 5-mm Jackson–Pratt wound drain (Baxter Healthcare, Deerfield, IL, USA) was left in the pouch of Douglas or rectovesical space and dedicated for peritoneal irrigation, usually performed on the first and fifth post-operative days. After clamping the other wound drains, 200 ml of warmed saline was infused via the pelvic drain, left in situ for 5 min and then allowed to drain back into the infusion bag.

### Specimen processing

All fluid samples were spun at 1500 r.p.m., the precipitate resuspended and then spun onto poly-L-lysine coated slides by centrifuge (Cytospin 2, Shandon Southern Productions, Runcorn, UK).

Slides from each sample were fixed and stained by Papanicolaou and Giemsa methods using routine automated cycles as well as manual periodic acid–Schiff staining with or without diastase incubation.

### Cytological interpretation

All slides were initially read by the principal author and were later second-read by a consultant cytopathologist (VW), who was unaware of the surgical or histological findings. Interobserver variation related to the assessment of staining was less than 5%. Discordant cases were re-evaluated by the two observers using double-headed light microscopy. In addition to the four stains described, specimens were examined using monoclonal antibodies to CK, CEA, p53, AUA 1 and B72 (data not presented). There were no instances in which standard techniques failed to identify malignant cells found in these latter techniques, thus we can be confident that the incidence of false-negative results because of post-collection sampling will be very low. Slide preparations were deemed inadequate if there was insufficient cellular material for diagnosis, if blood staining was so heavy that it prevented cellular examination, or if there had been a fault of fixation or staining. Malignant cells were recognized under light microscopy using standard criteria (Koss, 1992), namely by presence of large, hyperchromatic nuclei, abnormal nuclear chromatin, prominent nucleolus, distorted nuclear outline, increased nuclear/cytoplasmic ratio, intracytoplasmic acid or neutral mucin vacuoles, or heterogeneity of cells with clumping of groups. Each slide was classified as inadequate, benign, malignant or suspicious. The latter category was reserved for specimens in which scant solitary positive cells had been imperfectly fixed.

### Histological interpretation

The gastrectomy specimens were processed routinely and interpreted by a single experienced pathologist (DJS) blinded to operative and cytological findings according to a standardized dissection protocol. Specimens were staged according to the unified guidelines developed by the Union Internacionale Contra la Cancrum, the Japanese Research Society for Stomach Cancer and the American Joint Committee on Cancer (Kennedy, 1987).

### Follow-up

All patients were followed up for 2 years or until death. To ensure accurate survival data, the hospital notes were clearly labelled and the general practitioners informed of the nature of the study such that date of death was communicated promptly to the authors. Survival was confirmed by contact with general practitioners at 2 years.

### Statistical analysis

Continuous data was compared using the Mann–Whitney *U*-test, ordinal data by chi-squared test, and survival, calculated by the method of Kaplan–Meier, by the log-rank test. Significance was assumed if $P < 0.05$.

© Cancer Research Campaign 1999

**Table 1** Number of positive cytological cases (including cumulative incidence of new positive findings in addition to those detected by preresection washings) for each method of sampling

| | No. cases evaluated | Malignant cytology detected (+ 'suspicious' cases) | New cases of malignant cytology detected | Cumulative incidence of malignant cytology |
|---|---|---|---|---|
| Preresection | | | | |
| Lavage (including laparosocopy) | 85 | 16 (2) | 16 | 16 |
| Serosal brushing | 78 | 6 (9) | 4 | 20 |
| Subtotal | 85 | | | 20 |
| Post-resection | | | | |
| Imprint | 36 | 6 (3) | 2 | 22 |
| Lavage | 83 | 6 (4) | 1 | 23 |
| Subtotal | 85 | | | 23 |
| Post-operative | | | | |
| Irrigation 1 | 81 | 2 (2) | 0 | 23 |
| Irrigation 2 | 74 | 3 (3) | 0 | 23 |
| Total | 85 | | | 23 |

### RESULTS

### Patients

Eighty-nine patients were recruited in the 20-month period from April 1992. Four patients were excluded from analysis after unexpected post-resection pathological reports: two were discovered to have cancer arising in Barrett's oesophagus, two were discovered to have benign gastric ulceration despite pre-operative reports of high-grade dysplasia in a non-healing gastric ulcer. The median age of patients was 69 (range 32–95), 66 were men.

### Operative findings and procedures

Sixty-three of the 85 patients (74%) had macroscopic evidence of serosal penetration by tumour. Sixty-three had evidence of lymph node involvement. Twenty cases had evidence of peritoneal dissemination at the time of operation, five of whom had liver metastases also. A further three cases had liver metastasis without obvious peritoneal deposits.

Thirteen cases were unsuitable for resection and simple bypass was performed in five of these, 11 cases with advanced disease had palliative resections whereas the remainder underwent potentially curative D1 (47) or D2 (14) gastrectomy.

### Peritoneal cytology (Table 1)

Certain specimens were regarded as unreliable because either no cells at all or heavily bloodstained specimens were obtained. Inadequate specimens occurred in 9 out of 85 of preoperative peritoneal washes, 8 out of 83 post-resection washes, 11 out of 78 serosal brushings, 8 out of 36 imprint specimens, 0 out of 81 early post-operative irrigations and 1 out of 74 late post-operative irrigations.

In total, 23 patients (27%) had positive peritoneal malignant cytology. Of these cases, 22 out of 23 were observed to have serosal penetration by the primary tumour. Positive cytology was associated with a greater surface area of penetration [11.5 (7–14) vs. 5.5 (1–9) $cm^2$; $P < 0.05$]. There was no difference in sex ratio or age between the cytology-positive and-negative groups.

Peritoneal lavage alone yielded unequivocal malignant cytology in 16 out of 85 (19%) cases. Two patients with positive cytology at laparoscopy had inoperable disease and no further operative procedure was performed. Eight patients had ascites at the time of laparotomy or laparoscopy and all were shown to have free malignant cells. Serosal peritoneal brushings were positive in 6 of the 78 cases examined, four of these cases had been negative according to simple lavage. Imprint cytology yielded malignant cells in 6 of 36 cases, two had not been detected by simple lavage.

Eight patients with positive cytology underwent no operation or simple bypass procedure, six had palliative resection and nine had potentially curative (R0) resection. Of the nine patients with positive cytology who had undergone potentially curative resection, two were detected by preresection peritoneal lavage alone, two by imprint cytology alone, two by pre- and post-resection lavage and one by imprint cytology preresection and post-resection lavage. Malignant cells persisted post-resection in 9 of the 14 positive cases. In one case, preresection cytology was negative and post-resection cytology was positive.

**Table 2** Cytology status of patients with respect to T, N and M stage

| Stage | | Cytology Negative | Cytology Positive | Total |
|---|---|---|---|---|
| T* | T1 | 11 | 0 | 11 |
| | T2 | 25 | 1[a] | 26 |
| | T3 | 20 | 16 | 36 |
| | T4 | 6 | 6 | 12 |
| N** | N0 | 19 | 4 | 23 |
| | N1 | 30 | 12 | 42 |
| | N2 | 13 | 7 | 20 |
| M*** | M0 | 53 | 12 | 65 |
| | M1 | 9 | 11 | 20 |
| Total | | 62 | 23 | 85 |

*$\chi^2$ = 19.89; 3 d.f., $P$ < 0.001; **$\chi^2$ = 1.77; 2 d.f., $P$ = n/s (discrepancy with incidence of lymph node metastases described in operative findings suggest, overall, one case was over-staged by surgeon); *** $\chi^2$ = 10.35; 1 d.f., $P$ = <0.01 (discrepancy with incidence of metastases described in operative findings suggest, overall, three cases were over-staged by surgeon). [a]One patient had no serosal breach; cytology was negative before, but positive after, gastric resection.

© Cancer Research Campaign 1999

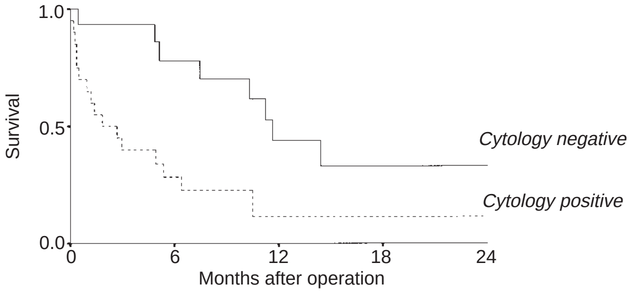

| | Start | 6 months | 12 months | 18 months | 24 months |
|---|---|---|---|---|---|
| Cytology negative | 26 | 20 | 13 | 10 | 6 |
| Cytology positive by lavage | 16 | 6 | 1 | 1 | 1 |
| by serosal brushings alone | 4 | 0 | 0 | 0 | 0 |
| by imprint cytology alone | 2 | 1 | 0 | 0 | 0 |

**Figure 1** Survival of patients with serosa-positive ($pT_3$, $pT_4$) gastric cancer according to cytology status, including analysis from brush and contact cytology (log-rank test = 8.44; 1 df, *P* = 0.004). Tabulation at foot of chart illustrates the contributions made by each sampling technique in identifying poor prognosis patients

### Histological interpretation (Table 2)

Positive cytological status was associated with significantly higher tumour (T) and metastasis (M) staging than the negative group, but no association was demonstrated between nodal (N) status and cytology.

### Survival

At 2-year follow-up, 22% of patients with positive cytology had survived, compared with 66% of the cytology-negative group (log-rank test = 25.10, 1 d.f., $P < 0.0001$). Favourable prognosis was also associated with a low TNM stage (log-rank test = 29.26, 3 d.f., $P < 0.0001$). Among patients who underwent a potentially curative (R0) resection, positive peritoneal cytology predicted poor survival (log-rank test = 10.8, 1 d.f.; $P = 0.001$). Peritoneal cytology from peritoneal lavage alone was not of prognostic significance in cases of T3 and T4, pathologically confirmed, serosa-positive disease (log-rank test = 0.33 1 d.f.; *P* = ns). Identification of cases by serosal brushings and imprint cytology in addition to cases detected by peritoneal lavage was associated with poor prognosis (log-rank test = 8.44; 1 d.f., $P = 0.004$) (Figure 1).

### DISCUSSION

Despite progress in recent years towards the early detection of gastric cancer (Sue-Ling et al, 1993), most patients will already have advanced disease at diagnosis (Allum et al, 1989*a*). The majority of patients will die of recurrent disease, even if surgery is thought to be curative at the time. A minority of patients with advanced gastric cancer have hitherto been shown to have free malignant cells at laparotomy (Nakajima et al, 1978; Boku et al, 1990; Bonenkamp et al, 1996). In the West, the success of peritoneal cytology has been limited. The Dutch Gastric Cancer Trial demonstrated positive cytology in only 7.1% of all patients with gastric cancer and 12% of cases with serosal invasion (Bonenkamp et al, 1996). The expected incidence of peritoneal metastasis suggests transcoelomic dissemination occurs more frequently than is demonstrated by cytology of preresection lavage fluid (Gunderson and Sosin, 1982).

In our study, post-resection peritoneal sampling was associated with a low yield of malignant cells. This may be explained to some extent by the changes associated with the surgical manoeuvre. In addition to blood staining, we found a considerable increase in the numbers of polymorphonuclear cells and mesothelial cells which change in appearance from regular, monotonous cells to bizarre, heterogeneous, multinucleated cells after surgery (Soosay et al, 1991). Any malignant cells remaining in the abdominal cavity after resection may be swamped by this population explosion of nucleated cells, making their detection more difficult. In addition, malignant cells rapidly adhere to either normal or denuded peritoneum. Surgical insult may contribute to this process by triggering the fibrin cascade which enmeshes malignant cells and facilitates their implantation (Sugarbaker et al, 1988). This too may explain the low yield of cells after resection.

Only one example occurred in which malignant cells could be identified post-operatively in a patient with a negative preresection status. However, in over half the cases with positive cytology on preresection examination, the subsequent samples were negative. The solitary patient in whom cytology status changed from negative to positive probably represents genuine cell dissemination that our techniques have failed to detect in an unknown proportion of other post-operative patients. This case, a T2 tumour, suggests that manipulation of the tumour can lead to peritoneal dissemination. Given that simple diagnostic lavage is an insensitive method for detecting free peritoneal cells, this single case is likely to understate the clinical problem of operative tumour dissemination. After therapeutic tetracycline or saline lavage, in only 3 of the 23 cases with positive cytology could free malignant peritoneal malignant cells be identified. This evidence supports the long-held surgical philosophy that careful tumour handling and adequate lavage at the end of cancer surgery may help to reduce the extent of malignant cell inoculation.

In our series, serosal brushings revealed relatively few positive cases despite histologically proven serosal penetration with tumour. The yield is less than one would expect if performing a brushing of the mucosal surface of the tumour. This might be explained by the reaction of adjacent mesothelial cells to the presence of tumour. Rapid healing of peritoneal wounds is well recognized, and it is likely that this phenomenon occurs to some extent when the visceral peritoneum is breached by tumour rather than by the scalpel. Bizarre mesothelial cell morphology was mainly encountered prior to resection in cases with tumour extending close to, or through, the serosal barrier.

In our series, 22 of 48 (46%) patients with locally advanced (pT3, pT4) cancer had positive peritoneal cytology. Although peritoneal lavage was the most sensitive single technique for demonstrating free peritoneal malignant cells, detecting malignant cells in 16 of 48 (33%) cases with locally advanced disease, the yield of free cells is significantly increased by the addition of both brush cytology and imprint cytology. By these methods, the incidence of positive peritoneal cytology begins to approach and hence predict the expected incidence of recurrence and death which may be as high as 80% (Gunderson and Sosin, 1982).

It has long been appreciated that patients with advanced gastric cancer offered surgery alone have little hope of being cured, and interest has recently been focused on the role of adjuvant therapy. Systemic chemotherapeutic agents have been investigated singly or in combination in randomized trials, but no therapy has yet been shown to be effective and the regimens are often associated with a high incidence of systemic side-effects (Allum et al, 1989*b*; Hallissey et al, 1994). Intraperitoneal chemotherapy administered as aqueous solution (Nakajima et al, 1978) or adsorbed onto carbon particles (Hagiwara et al, 1992) in the early post-operative period

*© Cancer Research Campaign 1999* 

appears to be well tolerated (Cunliffe and Sugarbaker, 1989). By this strategy, chemotherapeutic agents are delivered in maximum concentration to the site of maximum cancer cell contamination, at the time when the peritoneum is most vulnerable to implantation.

The peritoneal cytology status of a patient is an indicator of prognosis which can be determined rapidly using standard laboratory equipment and techniques. Although the role of adjuvant therapy for advanced gastric cancer requires further evaluation, peritoneal cytology may help to select those patients who have the most to gain from such treatment in the immediate perioperative period, before full detailed histological information is available.

In conclusion, addition of serosal brush cytology and imprint cytology to 'conventional' peritoneal lavage significantly increases the yield of free malignant peritoneal cells. Examination of free peritoneal malignant cells in this way predicts poor prognosis in cases of gastric cancer and among patients with advanced disease. Future studies evaluating the role of adjuvant treatments should take account of peritoneal cytological status, as well as conventional TNM staging criteria; this evaluation may have a prominent role in the selection of patients who have the most to gain from adjuvant therapy.

### ACKNOWLEDGEMENTS

This study was supported by grants from the Newcastle District Health Authority Scientific and Research Committee, and the Dr William Edmund Harker Foundation. We are indebted to Dr Peter Kelly for valuable advice on statistical analysis. The authors are grateful to the following consultants who permitted their patients to be included in this study: Mr WJ Cunliffe and Mr MJ Higgs (Queen Elizabeth Hospital, Gateshead), Mr MG Whittaker (Darlington Memorial Hospital), Mr TP Cole, Mr JG Palmer and the late Mr MJ Lyons (Cumberland Infirmary, Carlisle).

### REFERENCES

Allum WH, Powell DJ, McConkey CC and Fielding JWL (1989*a*) Gastric cancer: a 25-year review. *Br J Surg* **76**: 535–540

Allum WH, Hallissey MT, Ward LC and Hockey MS (1989*b*) A controlled, prospective, randomised trial of adjuvant chemotherapy or radiotherapy in resectable gastric cancer: interim report. *Br J Cancer* **60**: 739–744

Bonenkamp JJ, Songun I, Hermans J and van de Velde CJH (1996) Prognostic value of positive cytology findings from abdominal washings in patients with gastric cancer. *Br J Surg* **83**: 672–674

Boku T, Nakane Y, Minoura T, Takada H, Yamamura M, Hioki K and Yamamoto M (1990) Prognostic significance of serosal invasion and free intraperitoneal cancer cells in gastric cancer. *Br J Surg* **77**: 436–439

Cunliffe WJ and Sugarbaker PH (1989) Gastrointestinal malignancy: rationale for adjuvant therapy using early postoperative intraperitoneal chemotherapy. *Br J Surg* **76**: 1082–1090

Gunderson LL and Sosin H (1982) Adenocarcinoma of the stomach: areas of failure in a reoperation series (second or symptomatic look) clinicopathological correlation and implications for adjuvant therapy. *Int J Radiat Oncol Biol Phys* **8**: 1–11

Hagiwara A, Takahashi T, Kojima O, Sawai K, Yamaguchi T, Yamane T, Taniguchi H, Kitamura K, Noguchi A, Seiki K and Sakakura C (1992) Prophylaxis with carbon-adsorbed mitomycin against peritoneal recurrence of gastric cancer. *Lancet* **339**: 629–631

Hallissey M, Dunn J, Ward L and Allum W (1994) The second British Stomach Cancer Group trial of adjuvant radiotherapy or chemotherapy in resectable gastric cancer: five-year follow-up. *Lancet* **343**: 1309–1312

Iitsuka Y, Kaneshima S, Tanida O, Takeuchi T and Koga S (1979) Intraperitoneal free cancer cells and their viability in gastric cancer. *Cancer* **44**: 1476–1480

Kaibara N, Iitsuka Y, Kimura A, Kobayashi Y, Hirooka Y, Nishidoi H and Koga S (1987) Relationship between area of serosal invasion and prognosis in patients with gastric carcinoma. *Cancer* **60**: 136–139

Kennedy BJ (1987) The unified international gastric cancer staging classification system. *Scand J Gastroenterol* **22**(suppl. 133): 11–13

Koss LG (1992) Diagnostic cytology and its histopathologic basis, 4th edn., pp. 126–137. JB Lippincott: Philadelphia

Murphy PD, Wadhera V, Griffith SM, Burgess P, Farrell D, Taylor I, Hair T, Clague MB and Griffith CDM (1993) Free peritoneal cell identification in patients with gastric and colorectal cancer. *J R Coll Surg Edinburgh* **38**: 28–32

Nakajima T, Harashima S, Hirata M and Kajitani T (1978) Prognostic and therapeutic value of peritoneal cytology in gastric cancer. *Acta Cytol* **22**: 225–229

Papachristou DN and Fortner JG (1981) Local recurrence of gastric adenocarcinomas after gastrectomy. *J Surg Oncol* **18**: 47–53

Soosay GN, Griffiths M, Papadaki L, Happerfield L and Bobrow L (1991) The differential diagnosis of epithelial-type mesothelioma from adenocarcinoma and reactive mesothelial proliferation. *J Pathol* **163**: 299–305

Sue-Ling H, Johnston D, Martin IG, Dixon MF, Lansdown MRJ, McMahon MJ and Axon ATR (1993) Gastric cancer: a curable disease in Britain. *Br Med J* **307**: 591–596

Sugarbaker PH, Cunliffe W, Belliveau JF, De Bruijn EA, Graves T, Mullins R, Schlag P and Gianola F (1988) Rationale for perioperative intraperitoneal chemotherapy as a surgical adjuvant for gastrointestinal malignancy. *Reg Cancer Treat* **1**: 66–79

*© Cancer Research Campaign 1999*
